# Comparison of Dual Antiplatelet Therapies for Minor, Nondisabling, Acute Ischemic Stroke: A Bayesian Network Meta-Analysis

**DOI:** 10.1001/jamanetworkopen.2024.11735

**Published:** 2024-05-16

**Authors:** Andy Lim, Henry Ma, John Ly, Shaloo Singhal, Yuesong Pan, Yongjun Wang, S. Claiborne Johnston, Thanh G. Phan

**Affiliations:** 1 School of Clinical Sciences at Monash Health, Monash University, Melbourne, Victoria, Australia; 2 Department of Emergency Medicine, Monash Health, Melbourne, Victoria, Australia; 3 Department of Neurology, Division of Clinical Trials, Imaging, and Informatics, School of Clinical Sciences at Monash Health, Monash University, Melbourne, Victoria, Australia (SM); 4 University of California, San Francisco; 5 Harbor Health, Austin, Texas; 6 Department of Neurology, Beijing Tiantan Hospital, Capital Medical University, Beijing, China; 7 China National Clinical Research Centre for Neurological Diseases, Beijing, China

## Abstract

**Importance:**

Dual antiplatelet therapy (DAPT) appears to be an effective treatment option for minor (nondisabling) acute ischemic stroke. This conclusion is based on trials that include both transient ischemic attack (TIA) and minor stroke; however, these 2 conditions may differ.

**Objective:**

To compare DAPT regimens specifically for minor stroke.

**Data Sources:**

PubMed was searched for randomized clinical trials published up to November 4, 2023. Search terms strategy included *TIA*, *transient ischemic attack*, *minor stroke*, or *moderate stroke*, with the filter* randomized controlled trial*. Unpublished data on minor stroke were sourced from authors and/or institutions.

**Study Selection:**

Trials testing DAPT within the first 24 hours of a minor stroke (defined as a National Institutes of Health Stroke Scale score ≤5) were included by consensus. Of 1508 studies screened, 6 (0.3%) initially met inclusion criteria and were reviewed.

**Data Extraction and Synthesis:**

The study was performed according to Preferred Reporting Items for Systematic Reviews and Meta-Analyses guidelines by multiple observers. Bayesian fixed-effect network meta-analysis was conducted. Secondary analysis performed for high-risk TIA alone.

**Main Outcomes and Measures:**

Treatments were ranked using a probability measure called surface under the cumulative rank curve (SUCRA). The primary outcome was subsequent ischemic stroke at 90 days. Secondary outcomes included major hemorrhage, mortality, and hemorrhagic stroke. The number needed to treat (NNT) and number needed to harm (NNH) were obtained.

**Results:**

Five trials were included that described 28 148 patients, of whom 22 203 (78.9%) had a minor stroke. Of these, 13 995 (63.0%) were in DAPT groups and 8208 (37.0%) in aspirin (acetylsalicylic acid) groups. Aspirin and ticagrelor had a 94% probability of being the superior treatment for minor stroke (SUCRA, 0.94) for the primary outcome. Both aspirin and ticagrelor (NNT, 40; 95% CI, 31-64) and aspirin and clopidogrel (NNT, 58; 95% CI, 39-136) were superior to aspirin alone in the prevention of recurrent ischemic stroke at 90 days. Both treatments had higher rates of major hemorrhage than aspirin alone (NNH for aspirin and ticagrelor, 284; 95% CI, 108-1715 vs NNH for aspirin and clopidogrel, 330; 95% CI, 118-3430), but neither had increased risk of hemorrhagic stroke or death. For high-risk TIA, ticagrelor and aspirin had a 60% probability (SUCRA, 0.60) and clopidogrel and aspirin had a 40% probability (SUCRA 0.40) of being a superior treatment; neither was optimum, but both were superior to aspirin alone for the primary outcome.

**Conclusions and Relevance:**

These findings suggest that DAPT with aspirin and ticagrelor has higher probability of being the superior treatment among patients with minor stroke when presence of *CYP2C19* loss-of-function alleles has not been excluded. For patients with TIA, the superiority of aspirin and ticagrelor vs aspirin and clopidogrel was not demonstrated.

## Key Points

**Question:**

What is the optimum dual antiplatelet strategy for patients with minor, nondisabling, acute ischemic stroke?

**Findings:**

This meta-analysis of 5 dual antiplatelet therapy trials examining treatment for high-risk transient ischemic attack (TIA) and minor stroke among 28 148 patients showed that aspirin (acetylsalicylic acid) and ticagrelor had a 94% probability of being a superior treatment for minor stroke and a 60% probability as being the superior treatment for TIA.

**Meaning:**

These findings suggest that aspirin and ticagrelor has a higher probability of being the superior treatment among patients with minor stroke but not among those with TIA.

## Teaser

This network meta-analysis evaluates different dual antiplatelet therapy treatment regimens for patients with minor, nondisabling, acute ischemic stroke.

## Introduction

Minor (nondisabling) ischemic stroke, often defined as a National Institutes of Health Stroke (NIHSS) Scale score of 5 or less, comprises more than one-half of all acute ischemic stroke admissions.^1^ The optimal therapy for this condition has been defined recently with dual antiplatelet therapy (DAPT), and it has shown to be equivalent to alteplase.^2,3^ Currently, randomized clinical trials have included patients with both minor ischemic stroke and high-risk transient ischemic attack (TIA) and have shown that DAPT with either aspirin (ie, acetylsalicylic acid) and ticagrelor or aspirin and clopidogrel is superior to aspirin alone in preventing recurrent stroke.^4,5,6^ Both conditions are included in trials because they have similar prognosis, outcome, and presumed responses to antiplatelet therapy. However, there are possible reasons why the 2 conditions may differ, such as the higher chance of mimics (conditions that may be mistaken for an acute stroke or TIA [eg, migraine]) among the TIA group which would affect the rate of recurrence.^7,8,9^

We hypothesized that minor stroke has a different response to antiplatelet therapy when compared with TIA, owing to a higher grade of neurological injury and potentially higher risk of brain hemorrhage. Therefore, we sought to determine the optimum DAPT regimen in an enriched cohort with minor stroke. This study is different from a recent meta-analysis^17^ due to the inclusion of a newly published trial^10^ and availability of previously unpublished data from previous DAPT trials, provided by the authors.^4,5,11^ This study aims to compare the efficacy and safety of DAPT in patients with a minor, nondisabling, acute ischemic stroke using network meta-analysis (NMA) of clinical trials.^12^ To determine the differential effect of DAPT on minor stroke and high-risk TIA, we evaluated the effect of DAPT on high-risk TIA from these trials.

## Methods

### Eligibility Criteria

This meta-analysis conformed to the Preferred Reporting Items for Systematic Reviews and Meta-Analyses (PRISMA) Extension Statement for Reporting of Systematic Reviews Incorporating Network Meta-Analyses of Health Care Interventions.^13^ Articles included were randomized clinical trials of DAPT strategies within 24 hours of the onset of noncardioembolic minor ischemic stroke or high-risk TIA. Patients with minor ischemic strokes have a NIHSS score of 5 or less (scores range from 0 to 42, with higher scores indicating greater deficits). Ischemic stroke was defined as a new neurologic deficit lasting at least 24 hours that was not attributable to a nonischemic cause, or a new neurologic deficit not attributable to a nonischemic cause and accompanied by neuroimaging evidence of new brain infarction. High-risk TIA was defined in these trials as a score of 6 or 7 on the ABCD2 (age, blood pressure, clinical features, duration of transient ischemic attack, and diabetes) scale, which ranges from 0 to 7, with higher scores indicating higher risk of stroke. Stroke recurrence was defined as sudden onset of a new focal neurologic deficit with clinical or imaging evidence of infarction, or a rapid worsening of an existing focal neurologic deficit. Follow-up duration was 30 to 90 days.

### Information Sources

PubMed and reference lists of relevant papers were searched for studies published up to November 4, 2023. Search terms included *TIA*, *transient ischemic attack*, *minor stroke*, or *moderate stroke*, with the filter, *randomized controlled trial*. Full published papers and conference abstracts were considered. Reference lists of relevant articles and from experts in the field were involved in identifying relevant studies. Articles were included by consensus of the authors. If there were missing data in a relevant paper, the authors and/or the research institute (eg, National Institute of Neurological Disorders and Stroke) were contacted for unpublished data. Three study authors (Y.P., Y.W., and S.C.J) provided data from previously published studies.

Data included study name, author, cohort start and end years, publication year, upper limit of NIHSS inclusion, time to randomization in hours, cohort size, and number of events. The geometry of the network was described by a network diagram. Assessment of risk of bias within studies was performed using the Cochrane Risk of Bias tool for randomized trials.^14^

### Effect Measures

Treatment rankings were estimated with surface under the cumulative ranking curve (SUCRA) value,^15^ which is the probability that a treatment has of being the best option. The primary outcome measure was the risk of 90-day stroke recurrence (inclusive of both progression and recurrence); this was calculated as a proportion (number of events/cohort size) and reported as a percentage. Safety measures included the risk of hemorrhagic stroke, mortality, or major hemorrhage.

### Statistical Analysis

We performed Bayesian NMA using R statistical software version 4.3.1 with the BUGSnet (Bayesian Inference Using Gibbs Sampling to Conduct an NMA) and JAGS (Just Another Gibbs Sampler) packages (R Project for Statistical Computing).^16^ The BUGSnet library is recommended for NMA because it provides the necessary output required by various societies including the International Society of Pharmacoeconomics and Outcomes–Academy of Managed Care Pharmacy–National Pharmaceutical Council.^16^ NMA was used because of the ability to rank treatments using the SUCRA value.^15^ In this analysis, we used similar parameters for the analysis as employed by recent authors evaluating antiplatelet therapy,^17^ including a burn-in of 25 000 iterations (to allow the analysis to warm-up) followed by sampling of the next 50 000 iterations for analysis. Secondary analyses were conducted for TIA only, and for a combined minor stroke and TIA trial population from publicly available data. We conducted 2 sensitivity analyses; the first was to compare minor stroke outcomes at 30 days, and the second was to compare minor stroke outcomes at 90 days excluding data from 1 trial. Assessment of inconsistency, which refers to the agreement between indirect and direct evidence within a network,^18^ was tested using the inconsistency model,^16^ as recommended by the National Institute for Health and Clinical Excellence Decision Support Unit.^19^ The number needed to treat (NNT) and number needed to harm (NNH) were obtained using a mean control event rate calcuation^20^ similar to a previously published Bayesian NMA.^21^ Statistical significance was considered a 2-sided *P* < .05.

## Results

### Study Characteristics

**Figure 1.**
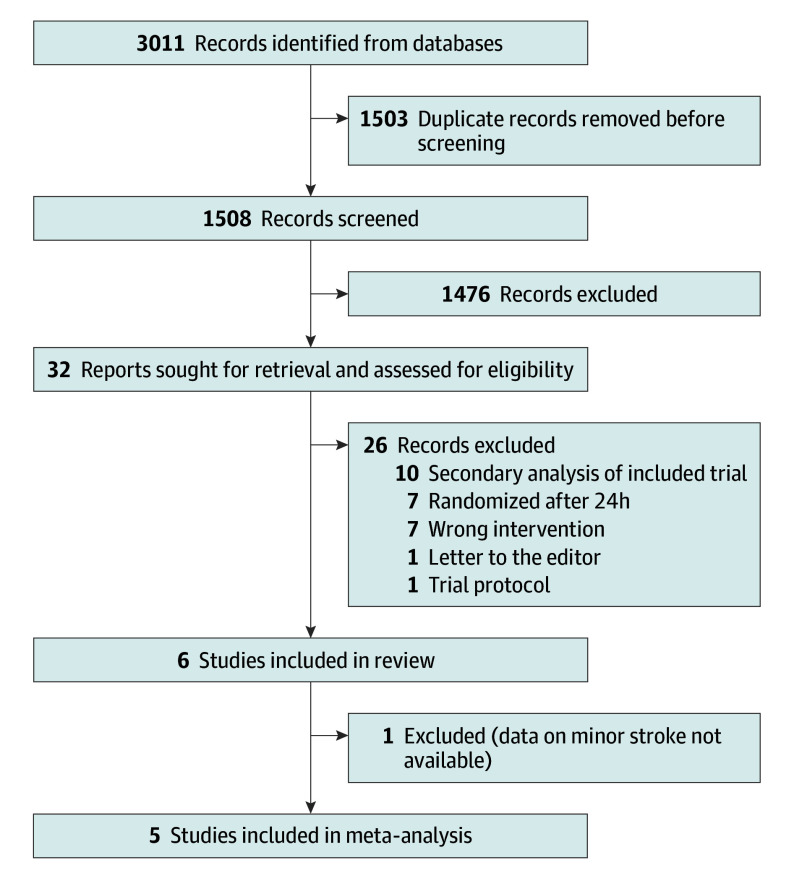
Preferred Reporting Items for Systematic Reviews and Meta-Analyses Flow Diagram

**Figure 2.**
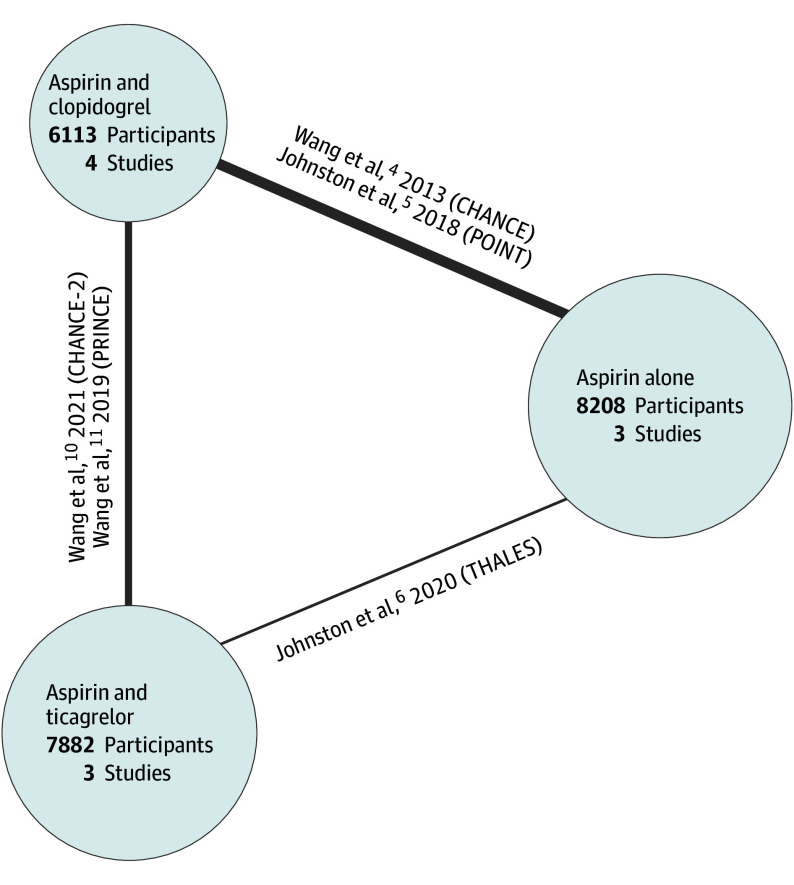
Network Diagram of Included Trials The sizes of the nodes are scaled to the number of studies examining a specific treatment. The thickness of the edges is scaled to the number of comparisons between treatments. Aspirin refers to acetylsalicylic acid. CHANCE indicates Clopidogrel in High-Risk Patients With Acute Nondisabling Cerebrovascular Events; CHANCE-2, Clopidogrel With Aspirin in High-Risk Patients With Acute Nondisabling Cerebrovascular Events II; POINT, Platelet-Oriented Inhibition in New Transient Ischemic Attack and Minor Ischemic Stroke; PRINCE, Platelet Reactivity in Acute Nondisabling Cerebrovascular Events; THALES, Acute Stroke or Transient Ischemic Attack Treated With Ticagrelor and Acetylsalicylic Acid for Prevention of Stroke and Death.

A total of 1508 records were screened and 6 (0.3%) initially met inclusion criteria for review (Figure 1). A total of 5 trials^4,5,6,10,11^describing 28 148 patients were included in the meta-analysis. Of all patients, 22 203 (78.9%) had a minor stroke. Of these patients, 13 995 (63.0%) were in DAPT groups and 8208 (37.0%) were in aspirin groups. We obtained unpublished pooled data for the minor stroke subset of existing trials for the 2013 Wang et al^4^ trial (ie, the Clopidogrel With Aspirin in Acute Minor Stroke or Transient Ischemic Attack [CHANCE] trial), the 2021 Wang et al^10^ trial (ie, CHANCE-2), and 2019 Wang et al^11^ trial (ie, the Platelet Reactivity in Acute Nondisabling Cerebrovascular Events [PRINCE] trial) from the trial authors, and individual patient data for the 2018 Johnston et al^5^ trial (ie, the Platelet-Oriented Inhibition in New Transient Ischemic Attack and Minor Ischemic Stroke [POINT] trial) from the National Institute of Neurological Disorders and Stroke. Characteristics of each trial are summarized in Table 1. The network plot is shown in Figure 2. Size of nodes are scaled to the number of studies examining a specific treatment. The thickness of edges is scaled to the number of comparisons between treatments.

**Table 1.** Study Characteristics of Dual Antiplatelet Therapy Trials in Minor Stroke

Source	Trial name	Cohort years	NIHSS range	Time to randomization, h	Treatment^a^	Aspirin, mg^a^		DAPT, mg		Treatment duration, d
						Loading	Maintenance	Loading	Maintenance	
Wang et al,^4^ 2013	CHANCE	2009-2012	0-3	24	Aspirin	75-300	75	NA	NA	21
Wang et al,^4^ 2013	CHANCE	2009-2012	0-3	24	Aspirin and clopidogrel	75-300	75	300	75	21
Johnston et al,^5^ 2018	POINT	2010-2017	0-3	12	Aspirin	50-325	50-325	NA	NA	90
Johnston et al,^5^ 2018	POINT	2010-2017	0-3	12	Aspirin and clopidogrel	50-325	50-325	600	75	90
Wang et al,^11^ 2019	PRINCE	2015-2017	0-3	24	Aspirin and clopidogrel	100-300	100	300	75	21
Wang et al,^11^ 2019	PRINCE	2015-2017	0-3	24	Aspirin and ticagrelor	100-300	100	180	90	21
Johnston et al,^6^ 2020	THALES	2018-19	0-5	24	Aspirin	300-325	75-100	NA	NA	30
Johnston et al,^6^ 2020	THALES	2018-2019	0-5	24	Aspirin and ticagrelor	300-325	75-100	180	90	30
Wang et al,^10^2021	CHANCE-2	2019-2021	0-3	24	Aspirin and clopidogrel	75-300	75	300	75	90
Wang et al,^10^2021	CHANCE-2	2019-2021	0-3	24	Aspirin and ticagrelor	75-300	75	180	90	90

Abbreviations: CHANCE, Clopidogrel in High-Risk Patients With Acute Nondisabling Cerebrovascular Events; CHANCE-2, Clopidogrel With Aspirin in High-Risk Patients With Acute Nondisabling Cerebrovascular Events II; DAPT, dual antiplatelet therapy; NA, not applicable; NIHSS, National Institutes of Health Stroke Scale; POINT, Platelet-Oriented Inhibition in New Transient Ischemic Attack and Minor Ischemic Stroke; PRINCE, Platelet Reactivity In Acute Nondisabling Cerebrovascular Events; THALES, Acute Stroke or Transient Ischaemic Attack Treated With Ticagrelor and Acetylsalicylic Acid for Prevention of Stroke and Death.

^a^ Aspirin refers to acetylsalicylic acid.

### Minor Stroke: Recurrent Stroke

Aspirin and ticagrelor consistently ranked first as the ideal therapy in X of Y simulations (94%; SUCRA, 0.94) (Table 2). Both aspirin and ticagrelor (hazard ratio [HR], 0.70; 95% credibility interval [CrI], 0.61-0.81) and aspirin and clopidogrel (HR, 0.79; 95% CrI, 0.69-0.91) were superior to aspirin alone in the prevention of recurrent ischemic stroke at 90 days. There was no statistically significant difference between the 2 combination treatments (HR, 1.13; 95% CrI, 0.97-1.31).

**Table 2.** Network Meta-Analysis Measures for Efficacy and Safety Up to 90 Days for Minor Stroke and for High-Risk TIA

Outcome measure^a^	Minor stroke (NIHSS ≤5)				High-risk TIA (ABCD^2^ >4)			
	Ischemic stroke	Hemorrhagic stroke	Mortality	Major hemorrhage	Ischemic stroke	Hemorrhagic stroke	Mortality	Major hemorrhage
SUCRA								
Aspirin and ticagrelor	0.94	0.15	0.75	0.00	0.60	0.08	0.03	0.00
Aspirin and clopidogrel	0.06	0.37	0.05	0.01	0.40	0.01	0.07	0.05
Aspirin alone	0.00	0.48	0.20	0.98	0.00	0.91	0.90	0.95
HR (95% CrI)								
Aspirin and ticagrelor vs aspirin alone	0.70 (0.61-0.81)	1.36 (0.54-3.53)	0.79 (0.45-1.41)	2.21 (1.20-4.19)	0.65 (0.43-0.98)	9.37 (0.44-848.72)	1.95 (0.98-3.99)	150.17 (4.66-214 774.80)
Aspirin and clopidogrel vs aspirin alone	0.79 (0.69-0.91)	1.06 (0.47-2.41)	1.18 (0.68-2.05)	2.04 (1.10-3.91)	0.68 (0.53-0.88)	7.37 (1.23-111.59)	2.06 (0.77-6.18)	2.20 (0.87-6.20)
Aspirin and ticagrelor vs aspirin and clopidogrel	0.89 (0.76-1.03)	1.28 (0.54-3.12)	0.68 (0.38-1.19)	1.08 (0.57-2.04)	0.95 (0.65-1.39)	1.24 (0.03-119.42)	0.94 (0.28-3.02)	66.52 (2.13-96 777.94)

Abbreviations: ABCD^2^, age, blood pressure, clinical features, duration of transient ischemic attack, and diabetes; CrI, credibility interval; HR, hazard ratio; NIHSS, National Institutes of Health Stroke Scale; SUCRA, surface under the cumulative rank curve; TIA, transient ischemic attack.

^a^ Aspirin refers to acetylsalicylic acid.

### Minor Stroke: Hemorrhagic Stroke

There was no significant increase in hemorrhagic stroke with both aspirin and ticagrelor (HR, 1.36; 95% CrI, 0.54-3.53) and aspirin and clopidogrel (HR, 1.06; 95% CrI, 0.47-2.41) (Table 2). Based on SUCRA values, aspirin alone was associated with the lowest risk of hemorrhagic stroke (SUCRA, 0.48), followed by aspirin and clopidogrel (SUCRA, 0.37), and aspirin and ticagrelor (SUCRA, 0.15) (Table 2).

### Minor Stroke: Mortality

There was no significant increase in mortality with both aspirin and ticagrelor (HR, 0.79; 95% CrI, 0.45-1.41) and aspirin and clopidogrel (HR, 1.18, 95% CrI, 0.68-2.05) compared with aspirin alone (Table 2). Aspirin and ticagrelor was associated with the lowest risk of mortality (SUCRA, 0.75), followed by aspirin alone (SUCRA, 0.20), and aspirin and clopidogrel (SUCRA, 0.05) (Table 2).

### Minor Stroke: Major Hemorrhage

Both aspirin and ticagrelor (HR, 2.20; 95% CrI, 1.21-4.19) and aspirin and clopidogrel (HR, 2.03; 95% CrI, 1.10-3.88) resulted in higher rates of major hemorrhage than aspirin alone (Table 2). Aspirin alone was associated with the lowest risk of hemorrhage (SUCRA, 0.98), followed by aspirin and clopidogrel (SUCRA, 0.01) and aspirin and ticagrelor (SUCRA, 0.00) (Table 2).

### High-Risk TIA

Secondary analysis was performed for high-risk TIA alone (ie, all the patients that were excluded from the main analysis). Neither regimen was considered favorable; aspirin and ticagrelor had a 60% probability of being the optimum treatment (SUCRA, 0.60) and aspirin and clopidogrel had a 40% probability of being the optimum treatment (SUCRA, 0.40) (Table 2). Both ASA and ticagrelor (HR, 0.65; 95% CrI, 0.43-0.98) and aspirin and clopidogrel (HR, 0.68; 95% CrI, 0.53-0.88) were superior to aspirin alone in the prevention of recurrent ischemic stroke at 90 days. There was no statistically significant difference between both combinations. Aspirin and clopidogrel appeared to increase risk of hemorrhagic stroke (HR, 7.37; 95% CrI, 1.23-111.59), and both aspirin and ticagrelor (HR, 150.17; 95% CrI, 4.66-214 774.80) and aspiring and clopidogrel (HR, 66.52; 95% CrI, 2.13-96 777.94) increased the risk of major hemorrhage.

### Combined Minor Stroke and High-Risk TIA

An analysis was also conducted on minor stroke and TIA combined, using the trials included in our study.^4,5,6,10,11^ Adding the additional 5945 patients with TIA to our minor stroke cohort of 22 203 patients did not make a clinically significant difference. Aspirin and ticagrelor was still the preferred regimen (SUCRA 0.92) compared with aspirin and clopidogrel (SUCRA 0.08), and both aspirin and ticagrelor (HR, 0.70; 95% CrI, 0.61-0.79) and aspirin and clopidogrel (HR, 0.77; 95% CrI, 0.68-0.87) were superior to aspirin alone for the primary outcome. Both aspirin and ticagrelor (HR, 2.89; 95% CrI, 1.62-5.42) and aspirin and clopidogrel (HR, 2.38, 95% CrI, 1.37-4.26) were associated with major hemorrhage when compared with aspirin alone (eTable 1 in Supplement 1).

Another analysis was conducted on minor stroke and TIA combined, using the same trials included in a previously published meta-analysis.^17^ This was different from our study because of the addition of the Fast Assessment Of Stroke and Transient Ischemic Attack to Prevent Early Recurrence (FASTER) trial by Kennedy et al,^22^ and the exclusion of the 2021 Wang et al^10^ trial (ie, CHANCE-2), which had not yet been published. Using this combination of trials, both aspirin and ticagrelor (HR, 0.76; 95% CrI, 0.65-0.89; SUCRA, 0.26) and aspirin and clopidogrel (HR, 0.71; 95% CrI, 0.62-0.82; SUCRA, 0.74) were superior to aspirin alone for the primary outcome. Both aspirin and ticagrelor (HR, 3.62; 95% CrI, 1.84-7.67) and aspirin and clopidogrel (HR, 2.13; 95% CrI, 1.26-3.72) were associated with increased risk of major hemorrhage when compared with aspirin alone (eTable 1 in Supplement 1).

### Sensitivity Analysis: 30-Day Recurrent Stroke, Hemorrhagic Stroke, Mortality, and Major Hemorrhage for Minor Stroke

Based on SUCRA values, aspirin and ticagrelor had the highest probability of being the best treatment option for the primary outcome (SUCRA, 0.93), followed by aspirin and clopidogrel (SUCRA, 0.07), and aspirin alone (SUCRA, 0.00) (eTable 2 in Supplement 1). Both aspirin and ticagrelor (HR, 0.72; 95% CrI, 0.62-0.82) and aspirin and clopidogrel (HR, 0.81; 95% CrI, 0.70-0.94) were superior to aspirin alone in the prevention of recurrent ischemic stroke at 30 days (eTable 2 in Supplement 1). There was no statistically significant difference between aspirin and ticagrelor compared with aspirin and clopidogrel at 30 days (HR, 0.88; 95% CrI, 0.75-1.04). Neither DAPT regimen was associated with an increase in hemorrhagic stroke or mortality. Both aspirin and ticagrelor (HR, 2.67; 95% CrI, 1.37-5.51) and aspirin and clopidogrel (HR, 3.11; 95% CrI, 1.42-7.30) were associated with an increase in major hemorrhage when compared with aspirin alone. Aspirin alone was associated with the lowest risk of major hemorrhage (SUCRA, 1.00) when compared with both DAPT regimens (SUCRA, 0.00) (eTable 2 in Supplement 1).

### Sensitivity Analysis: 90-Day Recurrent Stroke, Hemorrhagic Stroke, Mortality, and Major Hemorrhage for Minor Stroke, Excluding CHANCE-2

When the 2021 Wang et al^10^ trial (CHANCE-2) was excluded, aspirin and ticagrelor was no longer the superior combination for the primary outcome (SUCRA, 0.32) when compared with aspirin and clopidogrel (SUCRA, 0.68) (eTable 3 in Supplement 1). Both aspirin and ticagrelor (HR, 0.77; 95% CrI, 0.65-0.90) and aspirin and clopidogrel (HR, 0.73; 95% CrI, 0.62-0.85) were still superior to aspirin alone. Neither DAPT regimen was associated with a statistically significant increase in hemorrhagic stroke or mortality. Aspirin and ticagrelor was associated with an increase in major hemorrhage (HR, 2.71; 95% CrI, 1.35-5.82) compared with aspirin alone. Aspirin alone was associated with the lowest risk of major hemorrhage (SUCRA, 0.93) when compared with both DAPT regimens (eTable 3 in Supplement 1).

### Inconsistency, Risk of Bias, and NNT or NNH

Tests for inconsistency did not reveal large differences between direct and indirect estimates. Assessment of individual study bias demonstrated low risk overall (eTable 4 in Supplement 1). NNT, NNH, and calculated mean control event rates are provided in Table 3. When examining NNT and NNH, both aspirin and ticagrelor (NNT, 40; 95% CI, 31-64) and aspirin and clopidogrel (NNT, 58; 95% CI, 39-136) were superior to aspirin alone in the prevention of recurrent ischemic stroke at 90 days. Both had higher rates of major hemorrhage than aspirin alone (NNH for aspirin and ticagrelor, 284; 95% CI, 108-1715 vs NNH for aspirin and clopidogrel, 330; 95% CI 118-3430), but neither had increased risk of hemorrhagic stroke or death.

**Table 3.** NNT and NNH for Dual Antiplatelet Therapy in Minor Nondisabling Acute Ischemic Stroke at 90 Days

Outcome measure	Control event rate, mean, %	HR (95% CrI)		NNT (95% CI)	
		Aspirin and ticagrelor vs aspirin alone^a^	Aspirin and clopidogrel vs aspirin alone^a^	Aspirin and ticagrelor vs aspirin alone^a^	Aspirin and clopidogrel vs aspiring alone alone^a^
Minor stroke (n = 8208)					
Ischemic stroke	8.5	0.70 (0.61-0.81)	0.79 (0.69-0.91)	40 (31-64)	58 (39-136)
Hemorrhagic stroke	0.2	1.36 (0.54-3.53)	1.06 (0.47-2.41)	1359^b^	8151^b^
Mortality	0.4	0.79 (0.45-1.41)	1.18 (0.68-2.05)	1026^b^	1198^b^
Major hemorrhage	0.3	2.21 (1.20-4.19)	2.04 (1.10-3.91)	NNH (95% CrI), 284 (108-1715)	NNH (95% CRI), 330 (118-3430)
High-risk transient ischemic attack (n = 2320)					
Ischemic stroke	6.6	0.65 (0.43-0.98)	0.68 (0.53-0.88)	44 (27-783)	48 (33-130)
Hemorrhagic stroke	0.0	9.37 (0.44-848.72)	7.37 (1.23-111.59)	NNH, 378^b^	NNH (95% CI), 496 (29-13 724)
Mortality	0.9	1.95 (0.98-3.99)	2.06 (0.77-6.18)	113^b^	101^b^
Major hemorrhage	0.2	150.17 (4.66-214 774.80)	2.2 (0.87-6.20)	NNH (95% CI), 4 (1-134)	NNH, 409^b^

Abbreviations: CrI, credibility interval; HR, hazard ratio; NNH, number needed to harm; NNT, number needed to treat.

^a^ Aspirin refers to acetylsalicylic acid.

^b^ 95% CIs for these values are not presented because they were not statistically significant.

## Discussion

The key finding of this meta-analysis is that DAPT with aspirin and ticagrelor was almost always the superior treatment in the minor stroke subset of existing trials within our network simulation. This was mainly due to the inclusion of the 2021 Wang et al trial^10^(CHANCE-2), which was limited to patients with specific *CYP2C19* sequence variants that are associated with incomplete conversion of clopidogrel to its active form. We also showed that the superiority of aspirin and ticagrelor vs aspirin and clopidogrel was not demonstrated for patients with TIA. We achieved this by obtaining unpublished pooled data for the 2013 Wang et al^4^ trial (CHANCE), 2021 Wang et al^10^ trial (CHANCE-2), 2019 Wang et al trial^11^ (PRINCE), and 2018 Johnston et al^5^ trial (POINT) to perform the NMA analysis. These findings are important and challenge the current paradigm of assuming that minor stroke and high-risk TIA have similar response to antiplatelet therapy. Future trials and natural history studies need to consider these 2 conditions separately.

Our findings on different effectiveness of DAPT regimens for minor stroke needs to be considered in the context of the available data. Previous researchers have performed similar comparisons, but of pooled results from study populations that combine minor stroke and TIA.^17^ Using this approach, no regimen was clearly superior over another, with aspirin and clopidogrel being 66% most likely to be the superior treatment choice for reducing recurrent ischemic stroke (SUCRA, 0.66), and aspirin and ticagrelor being 34% most likely (SUCRA, 0.34).^17^ When we examined only TIA, we also found that there was no superior choice of DAPT combination. However, when we investigated only minor stroke, we found 1 DAPT regimen to be superior. A possibility is that the risk of stroke recurrence is higher with minor stroke than TIA and hence the effect of DAPT is more pronounced. It is possible that some of the patients in the high-risk TIA group had minor stroke, but the effect of DAPT may have been diluted by inadvertent inclusion mimics.^23^ An evaluation of UK TIA data^8^ revealed that the prevalence of mimics with ABCD^2^ (age, blood pressure, clinical features, duration of TIA, diabetes) score greater than 4 to be around 21.9%. What makes this group hard to discern from TIA is that 42.4% of mimics have some form of vascular disease including previous stroke, ischemic heart disease, or diabetes.^8^ The risk of subsequent stroke among this group is 0.19%.^8^

Our findings advance current understanding of DAPT therapy in minor stroke, as to our knowledge, no direct or indirect comparison has been made between aspirin and ticagrelor and aspirin and clopidogrel in nongenotyped populations. The 2 trials that compared the 2 treatments^10,11^ were performed in patients who carried *CYP2C19* loss-of-function alleles. Our finding is influenced by the addition of the 5000 patients with minor stroke patients from the Wang et al^10^ (CHANCE-2) trial, which demonstrated a lower 90-day stroke risk with ticagrelor than with clopidogrel, favoring the ticagrelor regimen. Aspirin and ticagrelor was no longer superior when we removed the patients from Wang et al^10^ from the analysis. This may also explain why previous research has not demonstrated superiority of ticagrelor because previous work^17^ did not include Wang et al.^10^

### Limitations

This study has limitations. First, minor stroke data from Kennedy et al^22^ could not be obtained, so this analysis does not contain all possible data. Second, generalizability of the results is limited due to the strict inclusion criteria of the included trials. For example, cardioembolic strokes and patients who required treatment more than 24 hours after symptom onset were excluded. Furthermore, the results are dependent on inclusion of a large group of patients with specific *CYP2C19* sequence variants that are associated with incomplete conversion of clopidogrel to its active form. Third, caution is warranted in interpreting our data because NMA calculates both direct and indirect comparisons within the network and does not represent an actual trial that compares the 2 DAPT regimens. Fourth, assessment of publication bias is difficult in an NMA due to the low number of studies in each comparison.^13^

## Conclusions

The findings of this meta-analysis suggest DAPT with ticagrelor and aspirin has a higher probability of being the superior treatment among patients with minor stroke when presence of *CYP2C19* loss-of-function alleles has not been excluded. This superiority was not demonstrated in patients with TIA. The findings reaffirm the need for DAPT to be used as a first-line treatment in patients with minor stroke due to noncardioembolic mechanisms.

## Notes

Supplement 1. **eTable 1.** Network Meta-Analysis Measures for Efficacy and Safety up to 90 Days for Both Minor Stroke and TIA Combined, Using Publicly Available Data **eTable 2.** Sensitivity Analysis for 30-Day Outcomes, Minor Stroke Only **eTable 3.** Sensitivity Analysis for 90-Day Outcomes, Minor Stroke Only, Excluding CHANCE-2 **eTable 4.** Quality Assessment

Supplement 2.
Data Sharing Statement
