# Supplementary material for: Comparison of Dual Antiplatelet Therapies for Minor, Nondisabling, Acute Ischemic Stroke: A Bayesian Network Meta-Analysis
Source: JAMA Netw Open. 2024 May 16;7(5):e2411735. doi: 10.1001/jamanetworkopen.2024.11735 (PMC11099682; doi:10.1001/jamanetworkopen.2024.11735)
Supplement: Supplement 1. — eTable 1. Network Meta-Analysis Measures for Efficacy and Safety up to 90 Days for Both Minor Stroke and TIA Combined, Using Publicly Available Data eTable 2. Sensitivity Analysis for 30-Day Outcomes, Minor Stroke Only eTable 3. Sensitivity Analysis for 90-Day Outcomes, Minor Stroke Only, Excluding CHANCE-2 eTable 4. Quality Assessment [file jamanetwopen-e2411735-s001.pdf]

Supplemental Online Content

Lim A, Ma H, Ly J, et al. Comparison of dual antiplatelet therapies in minor nondisabling acute ischemic stroke: a bayesian network meta-analysis. *JAMA Netw Open*. 2024;7(5):e2411735. doi:10.1001/jamanetworkopen.2024.11735

eTable 1. Network Meta-Analysis Measures for Efficacy and Safety up to 90 Days for Both Minor Stroke and TIA Combined, Using Publicly Available Data

eTable 2. Sensitivity Analysis for 30-Day Outcomes, Minor Stroke Only

eTable 3. Sensitivity Analysis for 90-Day Outcomes, Minor Stroke Only, Excluding CHANCE-2

eTable 4. Quality Assessment

This supplemental material has been provided by the authors to give readers additional information about their work.

© 2024 Lim A et al. *JAMA Network Open.*

eTable 1. Network Meta-Analysis Measures for Efficacy and Safety up to 90 Days for Both Minor Stroke and TIA Combined, Using Publicly Available Data

		Minor stroke and high-risk TIA combined (trials from the present study)		Minor stroke and high-risk TIA combined (trials from previously published meta-analysis)[17]	
	**Outcome measure**	Ischemic stroke	Major hemorrhage	Ischemic stroke	Major hemorrhage
**SUCRA**	**Ticagrelor + ASA**	0.92	0	0.26	0
	**Clopidogrel + ASA**	0.08	0	0.74	0
	**ASA**	0	1	0	1
**HR (95% CrI)**	**Ticagrelor + ASA vs ASA**	0.70 (0.61, 0.79)	2.89 (1.62, 5.42)	0.76 (0.65, 0.89)	3.62 (1.84, 7.67)
	**Clopidogrel + ASA vs ASA**	0.77 (0.68, 0.87)	2.38 (1.37, 4.26)	0.71 (0.62, 0.82)	2.13 (1.26, 3.72)
	**Ticagrelor + ASA vs Clopidogrel + ASA**	0.91 (0.79, 1.04)	1.21 (0.67, 2.20)	1.07 (0.87, 1.30)	1.70 (0.78, 3.90)

HR = hazard ratio

CrI = credibility interval

ASA = acetylsalicylic acid

© 2024 Lim A et al. *JAMA Network Open.*

eTable 2. Sensitivity Analysis for 30-Day Outcomes, Minor Stroke Only

	**Estimates from NMA**					
	**HR (95% CrI)**			**SUCRA**		
**Outcome measure**	**Ticagrelor and ASA vs ASA**	**Clopidogrel and ASA vs ASA**	**Ticagrelor and ASA vs Clopidogrel and ASA**	**Ticagrelor and ASA**	**Clopidogrel and ASA**	**ASA**
- Ischemic stroke	0.72 (0.62, 0.82)	0.81 (0.70, 0.94)	0.88 (0.75, 1.04)	0.93	0.07	0
- Hemorrhagic stroke	2.23 (0.82, 6.67)	2.09 (0.79, 5.94)	1.07 (0.40, 2.88)	0.05	0.06	0.89
- Mortality	0.81 (0.42, 1.52)	1.47 (0.67, 3.34)	0.55 (0.27, 1.09)	0.72	0.03	0.25
- Major hemorrhage	2.67 (1.37, 5.51)	3.11 (1.42, 7.30)	0.86 (0.40, 1.79)	0	0	1.00

© 2024 Lim A et al. *JAMA Network Open.*

eTable 3. Sensitivity Analysis for 90-Day Outcomes, Minor Stroke Only, Excluding CHANCE-2

	**Estimates from NMA**					
	**HR (95% CrI)**			**SUCRA**		
**Outcome measure**	**Ticagrelor and ASA vs ASA**	**Clopidogrel and ASA vs ASA**	**Ticagrelor and ASA vs Clopidogrel and ASA**	**Ticagrelor and ASA**	**Clopidogrel and ASA**	**ASA**
- Ischemic stroke	0.77 (0.65, 0.90)	0.73 (0.62, 0.85)	1.05 (0.85, 1.31)	0.32	0.68	0
- Hemorrhagic stroke	2.32 (0.79, 7.95)	0.79 (0.32, 1.91)	2.94 (0.88, 11.29)	0.02	0.69	0.29
- Mortality	0.94 (0.49, 1.78)	0.93 (0.51, 1.68)	1.00 (0.45, 2.26)	0.40	0.40	0.20
- Major hemorrhage	2.71 (1.35, 5.82)	1.69 (0.86, 3.44)	1.61 (0.66, 4.00)	0	0.07	0.93

© 2024 Lim A et al. *JAMA Network Open.*

eTable 4. Quality Assessment

Study	Year	Author	D1	D2	D3	D4	D5	Overall
CHANCE	2013	Wang	Low	Low	Low	Low	Low	Low
POINT	2018	Johnston	Low	Low	Low	Low	Low	Low
PRINCE	2019	Wang	Low	Low	Low	Low	Low	Low
THALES	2020	Johnston	Low	Low	Low	Low	Low	Low
CHANCE-2	2021	Wang	Low	Low	Low	Low	Low	Low

CHANCE = Clopidogrel in high-risk patients with acute non-disabling cerebrovascular events

POINT = Platelet-oriented inhibition in new TIA and minor ischemic stroke

PRINCE = Platelet reactivity in acute non-disabling cerebrovascular events

CHANCE-2 = Clopidogrel with aspirin in high-risk patients with acute non-disabling cerebrovascular events II

THALES = Acute STroke or Transient IscHaemic Attack Treated With TicAgreLor and ASA for PrEvention of Stroke and Death

© 2024 Lim A et al. *JAMA Network Open.*
