# Supplementary material for: Comparison of Dual Antiplatelet Therapies for Minor, Nondisabling, Acute Ischemic Stroke: A Bayesian Network Meta-Analysis
Source: JAMA Netw Open. 2024 May 16;7(5):e2411735. doi: 10.1001/jamanetworkopen.2024.11735 (PMC11099682; doi:10.1001/jamanetworkopen.2024.11735)
Supplement: Supplement 2. — Data Sharing Statement [file jamanetwopen-e2411735-s002.pdf]

Data Sharing Statement

Lim. Comparison of Dual Antiplatelet Therapies for Minor, Nondisabling, Acute Ischemic Stroke: A Bayesian Network Meta-Analysis. *JAMA Netw Open.* Published May 16, 2024. doi:10.1001/jamanetworkopen.2024.11735

Data
Data available: Yes
Data types: Deidentified participant data
How to access data: Both publicly available, or with the permission of individual authors.
When available: With publication

Supporting Documents
Document types: None

Additional Information
Who can access the data: Researchers whose proposed use of the data has been approved.
Types of analyses: Research purposes.
Mechanisms of data availability: With investigator support.
